# Microbial Shelf Life and Quality Assessment of Broiler Breast Meat: The Role of Cold Storage and Carcass Weight

**DOI:** 10.3390/foods14040640

**Published:** 2025-02-14

**Authors:** Abdullah Y. Abdullah, Anas Al-Nabulsi, Mohammad Jamama’h, Batool Khataybeh, Mu’ath Al-Ghadi

**Affiliations:** 1 Department of Animal Production, Jordan University of Science and Technology, Irbid 22110, Jordan; jamama86@yahoo.com; 2 Department of Nutrition and Food Technology, Jordan University of Science and Technology, Irbid 22110, Jordan; anas_nabulsi@just.edu.jo (A.A.-N.); khataibehbatool@gmail.com (B.K.); 3 Department of Zoology, College of Science, King Saud University, P.O. Box 2455, Riyadh 11451, Saudi Arabia; malghadi@ksa.edu.sa

**Keywords:** carcass weight, cold storage, fresh broiler, shelf life, spoilage rate, meat quality

## Abstract

Globally, poultry products have been associated with outbreaks of foodborne illnesses. The aim of this study was to evaluate the effects of cold storage period, carcass weight, and product form on fresh broiler bacteriology and meat quality parameters. A total of 500 one-day-old broiler birds were raised to market age (28–35 days) before slaughtering. The carcasses were classified into two groups: light weight (approximately 1100 ± 50 g) and heavy weight (approximately 1400 ± 50 g). After 4 h of post-chilling aging, 256 carcasses were randomly selected to represent the two categories. Each category of 128 carcasses was randomly distributed into two groups of 64 carcasses. One group was stored as whole carcasses, while the other group was stored as part-cut deboned breast meat at 4 °C for 1, 3, 5, and 7 days of cold storage (16 samples per storage day). Post-chilling temperature, pH, cooking loss, water holding capacity, and shear force were significantly affected by product form and storage period. Water holding capacity and shear force were also affected by carcass weight (*p* < 0.001). Meat colors (L*, a*, b*, chroma, and hue values) were significantly affected by the storage period. The L* value was only affected by product form and carcass weight (*p* < 0.01). Crude protein and ether extract were significantly affected by carcass weight and storage period, while ash was only affected by carcass weight. Aerobic plate count, psychrotrophic count, proteolytic count, lipolytic count, and coliform count were significantly increased with storage time. In conclusion, carcass weight had no impact on overall meat quality, but the meat began to deteriorate and showed an increased spoilage rate after five days of cold storage.

## 1. Introduction

Globally, the poultry sector is considered one of the most important economic sectors. Given this importance, developing and achieving sustainability in it is considered essential to achieving food security [1,2]. Poultry meat is a very popular food commodity around the world. In many countries, poultry consumption has risen, while beef and lamb consumption has declined over time [1,2]. Poultry is known for its high nutritional value, low fat content, relatively high levels of polyunsaturated fatty acids, and the wide variety of processed products available commercially, including fresh whole chickens and cut parts. Additionally, its relatively low production cost compared to red meat makes it an affordable and significant source of protein, contributing to its popularity [1,3,4,5]. The shelf life of fresh broiler meat, its microbiological safety, stability, and overall quality can be influenced by duration, chilling time, and chilling temperature [6]. The reduction in chilling temperature affected the physicochemical properties of carcasses, inhibited the growth of spoilage bacteria, and changed the structure of poultry meat [6]. Carcass weight may influence muscle structural characteristics, carcass composition, and broiler chicken meat qualities like color, tenderness, cooking loss, and water holding capacity [7,8,9]. The poultry industry is a cornerstone of global food production, providing a significant source of animal protein to meet the nutritional demands of a growing population. However, the industry faces persistent challenges, including various diseases that threaten flock health, productivity, and overall economic sustainability [10]. Food safety is of great public health concern since foodborne diseases are responsible for so many deaths and hospitalization annually in many different countries [11]. The “farm-to-fork” chain raises significant concerns regarding the inevitable contamination of animal meat [12]. Typically, the skin of animals can be tainted with fecal matter. There is a substantial risk of cross-contamination and the spread of microbes in slaughterhouses as well as during the handling, processing, and preparation stages [13]. The goal of food manufacturing today is to produce a stable, healthy, good-quality, and safe product that is free of foodborne pathogens and their toxins that are dangerous to a consumer’s health and that could lead to economic burdens [14,15,16,17]. Poultry meat has a higher pathogenic and spoilage bacterial count and is a highly perishable food that deteriorates easily after slaughtering, even under chilling temperature and storage conditions [14,18]. Most common pathogens found in poultry include *Salmonella* and *Campylobacter*, which are frequently present in the gastrointestinal tracts of birds [19]. Carcass contamination can occur at different stages of the poultry processing operation [20]. Fresh poultry meat shelf life is a function of the initial number of spoilage bacteria on the product’s surface and the period of time that the product is stored under refrigeration. Therefore, poultry meat safety must be controlled during processing from the beginning to the end, rather than relying on the detection of problems in finished poultry meat products. During cold storage, there are significant increases in the number of spoilage microorganisms in the fresh broiler chicken meat. Thus, meat is considered an expired product after 5–7 days of cold storage [21,22]. Food poisoning caused by spoiled poultry can be mild, with recovery in days, or severe, resulting in hospitalization and death in critically ill immunocompromised patients and in infants [23,24]. In Jordan, 60–70% of consumed meats are poultry meats [25]. Recently, several studies from Jordan have shown the spread of different types of foodborne pathogens in Jordanian poultry [26,27,28,29,30,31,32,33,34].

## 2. Materials and Methods

### 2.1. Experimental Procedure

A total of 500 one-day-old mixed-sex Lohman broiler birds were sourced from commercially hatched eggs. These chicks were raised under standard commercial practices until they reached market weight (1500–2200 g in a 35-day-long rearing period). According to the methodology outlined by Abdullah and Matarneh [35], the chicks were randomly distributed across 20 floor pens measuring 2 × 1.75 m^2^, with each pen housing 25 birds and lined with wood shavings. The pens were situated in an open-sided poultry house, each equipped with an automatic bell drinker and a tube feeder. The feeding regimen was designed to fulfill the nutritional requirements set by the NRC [36], with the diet primarily consisting of corn and soybean meal (Table 1).

### 2.2. Slaughter Procedure and Sampling

All birds were processed and slaughtered at 36 days of age following the procedures outlined by Abdullah and Matarneh [35]. At the end of the slaughtering process and at the grading stage, two types of carcass grade were chosen: category grade one was 1100 ± 50 g and category grade two was 1400 ± 50 g in a total of 256 carcasses (128 carcasses from each weight category). While all carcasses were stored in a chiller at 4 °C, postmortem internal muscle pH and temperature were recorded at 1, 2, 3, and 4 h using a knife incision on the right pectoralis major muscle. A portable pH meter and a digital thermometer were employed for these measurements, in line with the methods described by Abdullah and Matarneh [35]. After 4 h of post-chilling aging, each category of 128 carcasses was randomly distributed into two sub-groups of 64 carcasses. One sub-group was stored as whole carcasses, while the other group was processed as part-cut deboned breast meat. The deboning was carried out by a single individual to maintain consistency in the filet removal process, ensuring that the quality of the meat variables remain unaffected by the deboning technique. Subsequently, all samples were transported to the Meat Quality Laboratory at Jordan University of Science and Technology and then stored at 4 °C for 1, 3, 5, and 7 days of cold storage (16 samples per storage day for whole carcasses and deboned breasts) for further analysis.

### 2.3. Sample Preparation

At the end of each storage day, while the whole carcass group and the part-cut deboned breast meat group stored at 4 °C for 1, 3, 5, and 7 days of cold storage were still on their plates, the samples were taken out from refrigerator at each storage day and prepared for further analysis. The whole carcass samples were then cut up and deboned. Left and right pectoralis major muscles without skin were obtained from breast filets. For meat quality analysis, the left pectoralis muscle was chosen and used for measurements of cooking loss and tenderness, whereas the right pectoralis major muscle was used for color, pH, water holding capacity, water activity, microbial quality, and chemical composition measurements.

### 2.4. Meat Quality and Physicochemical Analysis of Breast Meat

#### 2.4.1. Cooking Loss

After breast deboning, each of the left pectoralis muscles were weighed (initial weight) and then placed in labeled polythene bags. The bags were placed in a thermostatically controlled water bath and cooked for 25 min at 85 °C to achieve a maximum internal temperature of 80 °C. After cooking, the bags were tempered at room temperature before opening to drain the liquid, and then the cooked sample was dried with a paper towel to remove excess surface moisture and re-weighed. Cooking loss was reported as the weight lost during cooking divided by the fresh sample weight and expressed as a percentage as previously described [35,37].

$$\%\;\mathrm{Cooking}\;\mathrm{loss}=100\;\times \;\frac{\left(Weight\;before\;cooking-Weight\;after\;cooking\right)}{Weight\;before\;cooking}$$

#### 2.4.2. Tenderness

Within 3 h of cooking, the dried samples from each of the left pectoralis major muscles were cut to obtain 6 cores (20 × 13 × 13 mm^3^) with similar sizes parallel to a line beginning at the humoral insertion and ending at the point adjacent to the keel and included the complete depth of each cooked muscle sample. Each core was sheared perpendicular to the longitudinal orientation of the muscle fiber using a Warner–Bratzler (WB) shear blade with the triangular slot cutting edge mounted on a Salter model 235 (Warner–Bratzler meat shear, G-R manufacturing Co., 1317 Collins LN, Manhattan, KS, USA) to determine the peak force (kg) when shearing the samples. Shear force was determined as the average of the maximum force of the six replicates from each pectoralis major muscle sample as previously described [35].

#### 2.4.3. Color

Instrumental color measurements of raw right pectoralis muscles were measured using a colorimeter (Color Tec-PCM Cole-Parameter International, Accuracy Microsensors Inc., Pittsford, NY, USA) calibrated throughout the study using a standard white ceramic reference (CIE L* = 97.91, a* = −0.68, b* = 2.45). Random readings were taken at 3 different locations on the muscle surfaces that were adjacent to the skin, in an area free of any noticeable color defects such as bruises or broken blood vessels in each sample. The 3 location readings were averaged, and the color of each sample was expressed in terms of CIE LAB (Commission International I E Calirage), wherein L*, a*, and b* represent the lightness, redness, and yellowness of the sample, respectively. Chroma C* and hue h° were calculated as described [37].

Chroma C* = (a*^2^ + b*^2^) and hue h° = tan^−1^(b*/a*).

#### 2.4.4. Water Holding Capacity

The water-binding properties of the breast pectoralis major muscles were estimated by measuring the amount of water released from the muscle protein by application of force (expressible juice) and by measuring the ability of the muscle protein to retain water present in excess and under the influence of internal force (water holding capacity (WHC).

After cutting each sample into small pieces, the sample meat pieces were covered with two filter papers (qualitative; 185 mm circular; fine crystalline retention; Whatman International Ltd., England) and two thin plates of quartz material and then pressed with a weight of 2500 g for 5 min. The meat samples were then removed from the filter paper, and their weight was recorded (final weight). The water holding capacity was reported as the weight lost during sample pressing divided by the initial sample weight and expressed as a percentage as described [37].

#### 2.4.5. Post-Storage pH

From 1 to 1.5 g of raw breast muscles was placed into a plastic test tube containing 10 mL of neutralized 5 mM iodoacetate reagent and 150 mM KCL and homogenized using a homogenizer (Ultra-Turrax T8, IKA Labortechnik, Janke and Kuunkal GmbH & Co., Staufen, Ltd., Germany). Before recording pH values of the solution on a pH meter (pH Spear, large screen, waterproof pH/temperature tester, double injection, model 35634-40, Eurotech instruments, Shah Alam, Malaysia), the electrode was rinsed with distilled water and dried with soft tissue paper.

#### 2.4.6. Water Activity Measurement

Water activity was measured by the Water Activity Meter (Aqualab, Pullman, WA, USA). Five grams of breast meat was placed in a small chamber, and then the water in the air was measured after it equilibrated with the sample [16].

### 2.5. Proximate Analysis of Broilers Breast Muscle Meat

Samples of breast muscle meat of light and heavy carcass weight were taken to be chemically analyzed. Samples were dried at 100 °C in a forced-air oven to reach a constant weight, air equilibrated, and then ground by a Wiley mill to pass a 1 mm screen (Barbender OHG Duisdurg, Kulturstrase 51-55, type 880845, Nr. 958084, Duisburg, Germany). Proximate analysis was performed on the raw breast meat according to AOAC [38] for dry matter (100 °C for 24 h; method 967.03), ash (550 °C for 8 h; method 942), crude protein (Kjeldahl procedure; method 976.06), and total lipid (ether extraction procedure; Soxhlet procedure, method 920.29).

### 2.6. Microbiological Analysis of Whole Carcasses and Deboned Breast Meat

For microbiological analysis, total aerobic count was determined using plate count agar (PCA, Oxoid Ltd., Basingstoke, UK) incubated at 35 °C for 24 h and at 7 ± 1 °C for 10 days for psychrotrophic count (PTC). The proteolytic count (PLC) was evaluated using Skim Milk Agar containing 10% skim milk (HiMedia, Ltd., Mumbai, India) incubated at 21 ± 1 °C for 3 days. Colonies with adjacent zones of proteolysis were counted as proteolytic bacteria. Spirit Blue Agar (HiMedia, Ltd., Mumbai, India) was used for lipolytic count (LLC) enumeration at 30 ± 1 °C for 3 days. Lipolytic cells had a dark blue area beneath and around them. Violet Red Bile Agar (VRBA, HiMedia, Ltd., Mumbai, India) was used for coliform count (CC) enumeration at 37 °C for 48 h. The enumeration of these microorganisms was evaluated at intervals of 1, 3, 5, and 7 days.

### 2.7. Statistical Analysis

The data were analyzed as 2 × 2 × 4 factorial designs (complete randomized design) using the statistical analysis and GLM procedure of SAS [39]. Data were analyzed by three-way ANOVA, with broiler carcass weight, product form, and storage period as the main effects. The least square means were calculated for all variables in the study, and the related LSD was calculated to determine significant differences. Differences were considered significant at *p* ≤ 0.05. The data were analyzed according to the following statistical model:

Yijk1= µ + Wi + Sj + Ak + (WS)ij + (WA)ik + (SA)jk + (WSA)ijk + Eijk

where µ = population mean; Wi = effect of product uniform (i = 1–2); Sj = effect of carcass weight (j = 1–2); Ak = effect of storage period (k = 1–4); (WS)ij, (WA)jk, (SA)jk, and (SA)ijk = interactions of the main effects; and Eijk1 = overall error term.

## 3. Results

### 3.1. Effects of Carcass Weight and Postmortem Time on Carcass Temperature and pH as Affected by Postmortem Time (PT) and Carcass Weight (CW)

**Figure 1:**
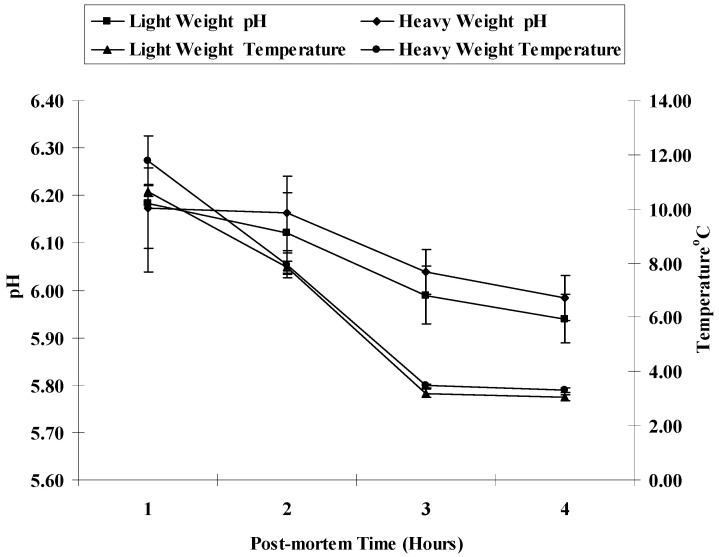
Carcass pH and temperature as affected by postmortem time in broiler chicken slaughtered at different carcass body weights.

Table 2 and Figure 1 show the influence of carcass weight and postmortem time on postmortem temperature and pH measurements recorded during the rigor mortis process. There were no significant differences between heavy and light carcass weights in pectoralis major muscle pH and temperature. Table 2 and Figure 1 also showed the pH and temperature decline during 1, 2, 3, and 4 h of postmortem time. Postmortem pH was significantly (*p* < 0.037) decreased by postmortem time. There were no significant differences in the postmortem pH values at 1, 2, and 3 h, but a postmortem time of 4 h showed a significantly lower pH value. Carcass temperature was also significantly (*p* < 0.0001) decreased by postmortem time. A gradual decrease in temperature was observed during rigor mortis development with no differences observed after 3 h postmortem.

### 3.2. Effects of Product Form, Carcass Weight, and Storage Period on Meat Post-Chilling Temperature, Cooking Loss, Water Holding Capacity, and Shear Force

The influence of product form, carcass weight, and storage period on post-chilling temperature, pH, cooking loss, water holding capacity and Warner–Bratzler shear force values of the breast meat are presented in Table 3.

#### 3.2.1. Effects of Product Form, Carcass Weight, and Storage Period on Meat Post-Chilling Temperature

**Figure 2:**
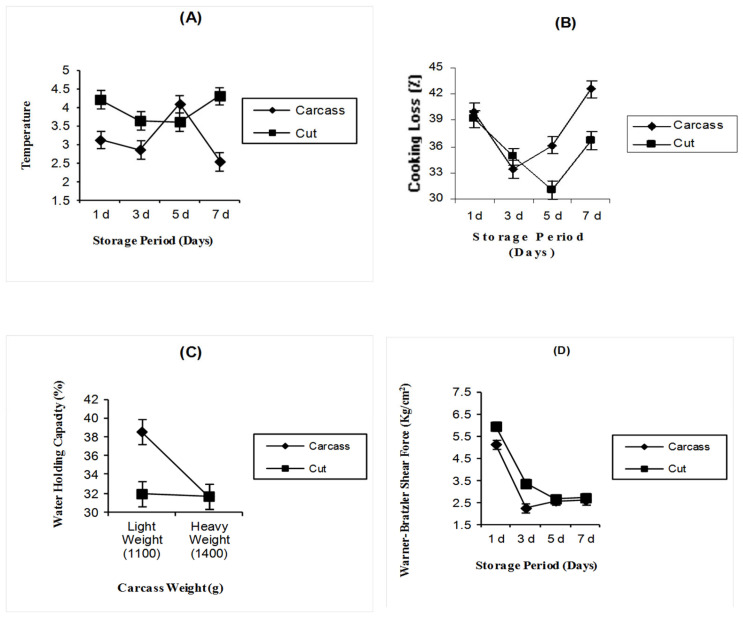
(**A**) The interactions of temperature, (**B**) cooking loss, and (**D**) Warner–Bratzler shear force for whole carcasses and part-cut deboned breast meat in relation to changes in storage period and the interaction of water holding capacity for whole carcasses and part-cut deboned breast meat in relation to changes in carcass weight (**C**).

Temperature was significantly affected by product form (*p* < 0.0001), and temperature was significantly (*p* < 0.0001) affected by the interaction between product form and storage period (Figure 2A). Post-chilling temperatures for part-cut deboned breast meat were higher at days 1, 3, and 7 than whole carcass, but at day 5, whole carcass post-chilling temperature reached a higher value than that for deboned breast meat.

The post-chilling pH values presented in Table 3 were not significantly affected by product form or carcass weight but were significantly (*p* < 0.0001) affected by storage period.

#### 3.2.2. Effects of Product Form, Carcass Weight, and Storage Period on Meat Cooking Loss

There is a highly significant (*p* < 0.0005) difference in cooking loss between whole carcasses and part-cut deboned breast meat. Cooking loss in whole carcasses was significantly higher than in deboned breast meat (38.01 vs. 35.43) (Table 3). Cooking loss values were not significantly affected by carcass weight. Carcass weight in this experiment did not differ enough to alter breast meat muscle cooking loss. There were significant (*p* < 0.0001) differences in cooking loss as affected by storage period (Table 3). Breast meats that were stored for 1 and 7 days had a significantly higher cooking loss than breast meats stored for 3 and 5 days. Table 3 and Figure 2B show the cooking loss as affected by the interaction of product form and storage period (*p* < 0.0001). On days 1, 5, and 7, the whole carcasses had a higher cooking loss than that from part-cut deboned breast meat, but at day 3, deboned breast meat had a higher cooking loss than that from the whole carcasses.

#### 3.2.3. Effects of Product Form, Carcass Weight, and Storage Period on Meat Water Holding Capacity

The water holding capacity (WHC) of fresh broiler chicken meat has an economic value because it affects the product profitability and sensory properties of the meat. As the WHC was measured by the value of expressible juiciness, the lower expressible juiciness value indicated the higher ability of meat to retain water. Table 3 showed that WHC% was significantly (*p* < 0.015) affected by product form. The percentage of whole carcasses (33.74) means that the carcasses can hold less water than part-cut deboned breast meat (30.72). The WHC of chilled breast meat decreased significantly (*p* < 0.0461) with increasing storage time (Table 3). A similar WHC was shown for chilled broiler breast meats that were stored for 1, 3, and 5 days, but 7 days of cold storage had the lowest WHC. Water holding capacity was significantly (*p* < 0.0008) affected by the interaction between carcass weight and product form (Figure 2C). Whole carcass from the lightweight group had a higher WHC than those from the part-cut deboned breast meat group; however, whole carcasses and deboned breast meat from the heavy weight group had a similar WHC.

#### 3.2.4. Effects of Product Form, Carcass Weight, and Storage Period on Meat Shear Force

Table 3 presents shear force values as kilograms per square centimeter (kg/cm^2^) with the higher shear force indicating less tender meat. Tenderness was significantly (*p* < 0.0004) affected by product form. Whole carcass meat had significantly lower shear force than deboned breast meat, with mean values of 3.14 and 3.67 kg/cm^2^ for whole carcass and deboned breast meat, respectively. Shear force was significantly (*p* < 0.0005) affected by carcass weight (Table 3). A heavy carcass weight resulted in significantly higher shear force values (3.66 kg/cm^2^) compared with light carcass weights (3.15 kg/cm^2^). Tenderness increased significantly (*p* < 0.0001) with an increasing storage period. The means of shear force values for storage periods 3, 5, and 7 were not statistically different from each other but had significantly lower shear force values than the day 1 storage period. A significant (*p* < 0.029) product form–storage period interaction was observed for shear force values (Figure 2D). Shear force values were higher for part-cut deboned breast meat than for whole carcasses throughout the entire storage period.

### 3.3. Effects of Product Form, Carcass Weight, and Storage Period on Meat Color Coordinate Values

**Figure 3:**
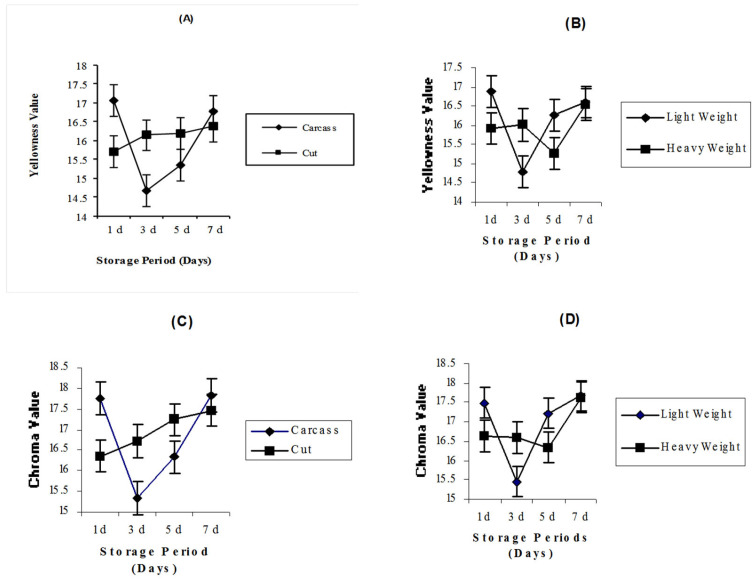
(**A**) The interactions of yellowness value and chroma value (**C**) for whole carcass and part-cut deboned breast meat in relation to changes in storage period. (**B**) The interaction of yellowness and chroma value (**D**) for light and heavy carcass weights in relation to changes in storage periods.

The least-squares means of broiler breast meat color as affected by product form, carcass weight, and storage duration are shown in Table 4. The lightness value was significantly (*p* < 0.05) affected by product form, whereas redness, yellowness, chroma, and hue were unaffected (Table 4). The whole carcass had higher lightness values (52.84) than deboned breast meat (51.71). There were no significant differences in breast meat redness, yellowness, chroma, and hue due to the change in carcass weight, but the lightness value was also significantly (*p* < 0.005) affected by carcass weight (Table 4). The meat with the highest lightness (L*) appears in the light carcass weight compared with the heavy carcass weight. The mean L* values were 52.98 and 51.57 for light and heavy carcass weights, respectively. Overall, chilled carcasses tended to be lighter and showed less redness and less yellowness in 1 and 3 days of cold storage compared with 5 and 7 days, which were characterized by being darker, redder, and more yellow. Lightness values were significantly decreased with increasing storage time (*p* < 0.0001). Table 4 also showed that redness values significantly increased with increasing storage period (*p* < 0.0001), whereas day 3 had the lowest redness value and day 7 had the highest value (redder, darker). The yellowness value was significantly affected by storage period (*p* < 0.05). The lowest yellowness value was observed on day 3 significantly differing from the breast meat stored for 1 and 7 days. Yellowness values were affected (*p* < 0.05) by the interaction between carcass weight and storage period (Figure 3B). On day 1 and 5, lighter weights had a higher yellowness value than those from heavier weights, whereas on day 3, lighter weights had a lower yellowness value than those from heavier weights, but at day 7, both weights had similar yellowness values. A significant (*p* < 0.01) interaction was also observed between product form and storage period for yellowness values (Figure 3A). Whole carcasses stored for 1 and 7 days showed a higher yellowness value than those for part-cut deboned breast meat, whereas part-cut deboned breast meat at day 3 and 5 had a higher yellowness value than whole carcasses. The chroma value was significantly (*p* < 0.001) affected by storage period (Table 4). Chroma values for breast meat stored for 1 day were similar to those stored for 5 days but differed from breast muscle stored for 3 and 7 days, with the lowest value registered on day 3 and the highest value recorded on day 7. A significant (*p* < 0.01) product form–storage period interaction was shown for chroma values (Figure 3C). The whole carcasses stored for 1 and 7 days showed a higher chroma value than those for deboned breast meat as part-cut, whereas part-cut deboned breast meat at day 3 and 5 had higher chroma values than the whole carcass values. Chroma values were also significantly affected (*p* < 0.05) by the interaction between carcass weight and storage period (Figure 3D). On day 1 and 5, lighter weights had higher yellowness values than those from heavier weights, whereas at day 3 lighter weights had a lower yellowness value than those from heavier weights, but on day 7 both weights had similar yellowness values. The hue value was significantly decreased with storage period (*p* < 0.0001), whereas breast meat stored for 1 and 3 days and for 5 and 7 days had similar values.

### 3.4. Effects of Carcass Weight and Storage Period on Chemical Compositions of Broiler Breast Meat

Table 5 shows the chemical composition of broiler breast meat stored at different storage periods within different carcass weights. Moisture% was not significantly affected by carcass weight, but crude protein, ether extract, and ash% were significantly affected by carcass weight. Crude protein% (*p* < 0.05) and ash% (*p* < 0.05) were significantly higher in light weight carcasses compared with heavy weight carcasses. Ether extract% was significantly higher (*p* < 0.0001) in heavy weight (1.0%) than light weight (0.70%) groups. The results for crude protein% showed that the breast meat stored for 1, 3, and 5 days did not differ significantly. However, breast meat stored for 7 days of cold storage showed significantly higher crude protein% compared with 1, 3, and 5 days. A similar trend was observed for ether extract.

### 3.5. The Effect of Cold Storage on Bacteriological Quality and Shelf Life of Fresh Whole Carcass and Deboned Broiler Breast Muscle Meat

Microbial populations (log_10_ CFU/g) of APC, LLC, PLC, PTC, and CC increased significantly during cold storage, following the order APC > LLC > PLC > PTC > CC throughout the storage period. Water activity remained consistent at 0.991 for both deboned breast meat and whole carcass.

**Figure 4:**
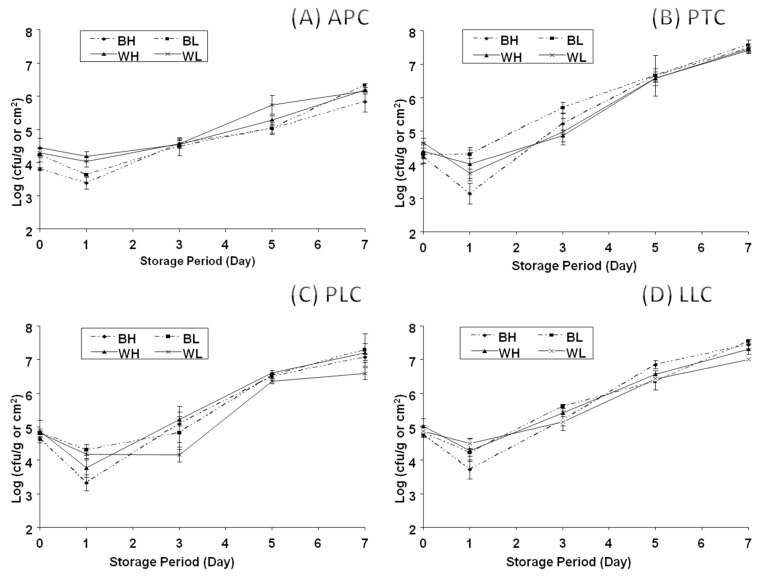
Effects of cold storage periods on: (**A**) aerobic plate count (APC); (**B**) psychrotrophic count (PTC); (**C**) proteolytic count (PLC); and (**D**) lipolytic count (LLC) of whole carcass and part-cut deboned breast meat during storage. Values represent the means ± SE of three samples, and each sample was measured in duplicate (n = 6 per treatment). BH: deboned breast meat from heavy carcasses (1400 ± 50 g). BL: deboned breast meat from light carcasses (1100 ± 50 g). WH: whole carcass from heavy carcasses (1400 ± 50 g). WL: whole carcass from light carcasses (1100 ± 50 g).

Figure 4 shows aerobic plate count (APC) of whole carcasses and deboned breast meat for heavy and light carcass weights at different cold storage periods (1, 3, 5, and 7 days). The initial microbial load of BH (deboned breast meat from heavy carcasses (1400 ± 50 g)) and BL (deboned breast meat from light carcasses (1100 ± 50 g)) was 3.81, 4.23 log_10_ CFU/g, respectively, while it was 4.46 and 4.29, log_10_ CFU/g in WH (whole carcass from heavy carcasses (1400 ± 50 g) and WL (whole carcass from light carcasses (1100 ± 50 g)), respectively. A significant reduction in the viability of aerobic bacteria was noted after the first day of chilled storage. Unpredictably, the APC was lower in BH and BL (3.38 and 3.63, log_10_ CFU/g, respectively) than WH and WL of APC (4.19 and 4.03 log_10_ CFU/g, respectively).

The APC increased in whole carcasses more than in deboned breast meat (Figure 4A). This could be explained by the larger surface area and availability of nutrients in whole carcasses that could support the growth of spoilage microorganisms. Figure 4B presents the psychrotrophic count (PTC) as affected by different cold storage times (1, 3, 5, and 7 days). The initial PTC after slaughtering for BH and BL was 4.22 and 4.31 log_10_ CFU/g, respectively, and the values for WH and WL were 4.41 and 4.65 log_10_ CFU/g, respectively.

Figure 4C presents the proteolytic count (PLC) as affected by different cold storage times (1, 3, 5, and 7 days). The initial proteolytic count of BH and BL increased from 4.75 and 4.81 log_10_ CFU/g to 7.07 and 7.29 log_10_ CFU/g, respectively, after 7 days of cold storage. Similarly, the initial proteolytic count of WH and WL increased from 4.86 and 4.82 to 7.20 and 6.58 log^10^ CFU/g, respectively, after 7 days of cold storage. Figure 4D presents the lipolytic count (LLC) as affected by different cold storage time (1, 3, 5, and 7 days). The initial lipolytic count of BH and BL was 4.75 and 4.81 log_10_ CFU/g, respectively, while it was 5.02 and 4.87 log_10_ CFU/g in WH and WL, respectively. The lipolytic count increased to 7.44 and 7.53 log_10_ CFU/g in BH and BL, respectively, and 7.31 and 7.01 log_10_ CFU/g in WH and WL, respectively, after 7 days of storage. It is obvious that the lipolytic count increased beyond the spoilage detection limit after 7 days of storage at 4 °C.

**Figure 5:**
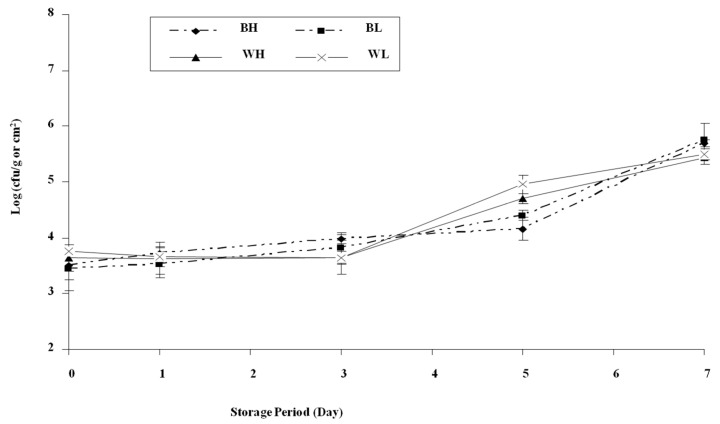
Effects of cold storage periods on the coliform count (CC) of whole carcasses and part-cut deboned breast meat during storage. Values represent the means ± SE of three samples, and each sample was measured in duplicate (n = 6 per treatment). BH: deboned breast meat from heavy carcasses (1400 ± 50 g). BL: deboned breast meat from light carcasses (1100 ± 50 g). WH: whole carcass from heavy carcasses (1400 ± 50 g). WL: whole carcass from light carcasses (1100 ± 50 g).

Figure 5 shows the coliform count (CC) at different cold storage times (1, 3, 5, and 7 days). The initial coliform count after slaughtering (Day 0) in BH, BL, WH, and WL (log_10_ CFU/g) were 3.5, 3.44, 3.64, and 3.75, respectively. As cold storage proceeds to 7 days, a slightly significant difference in coliform count between whole carcasses and deboned breast meat was noted. During 7 days of storage, the coliform count increased to reach 5.69 and 5.76 log_10_ CFU/g for BH and BL, respectively, and in WH and WL it was 5.44 and 5.49 log_10_ CFU/g, respectively.

**Figure 6:**
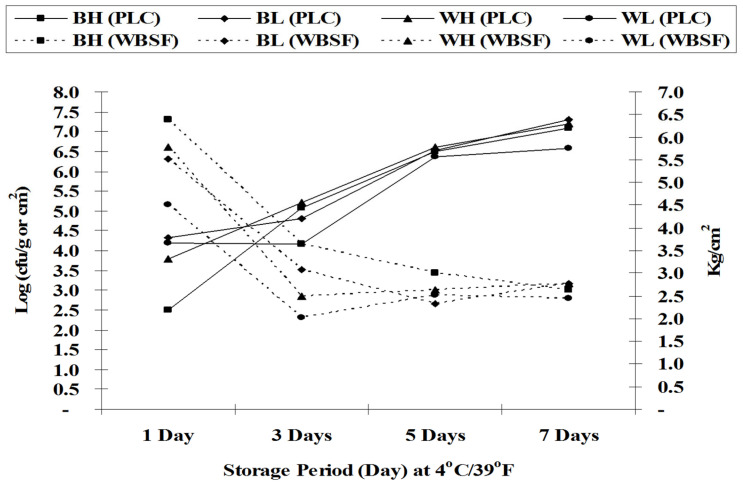
The correlation between proteolytic count (PLC) and Warner–Bratzler shear force (WBSF) as affected by cold storage periods. Values represent the means ± SE of three samples, and each sample was measured in duplicate (n = 6 per treatment). Warner–Bratzler shear force in kilograms per centimeters squared. BH: deboned breast meat from heavy carcasses (1400 ± 50 g). BL: deboned breast meat from light carcasses (1100 ± 50 g). WH: whole carcass from heavy carcasses (1400 ± 50 g). WL: whole carcass from light carcasses (1100 ± 50 g).

Figure 6 presents the correlation between proteolytic count (PLC) and Warner–Bratzler Shear Force (WBSF) during cold storage. In general, as cold storage time increased, PLC increased, WBSF decreased, and the tenderness of meat increased. At the end (Day 7) of cold storage for both whole carcasses and deboned breast meat, spoilage signs such as sliminess, off-odors, discoloration by destruction of meat pigments or growth of colonies of colored organisms, taint production, and fat decomposition were noted.

## 4. Discussion

The alteration in muscle pH is a crucial change that happens during rigor mortis and can significantly influence various quality attributes of meat, including texture, color, and water retention capacity. The duration for the rigor mortis process to fully complete differs based on factors such as species, muscle type, fiber type, storage temperature, and the level of agitation experienced at the time of death [8]. However, among those factors, muscle temperature appears to be more influential in the rate and extent of postmortem pH decline. There were no significant differences between heavy and light carcass weights in pectoralis major muscle pH and temperature at the end of rigor mortis time. This may be due to small differences between the two chosen weights and to small changes in muscle postmortem metabolism between the two carcass weights, which resulted in minimal differences in the rate of pH decline (Table 2 and Figure 1). However, the pH and temperature decline during 1, 2, 3, and 4 h of postmortem time. Similar results were previously reported [35,40,41] in that birds of different live body weights did not differ in their means of pH or their rate of postmortem decline of the pectoralis major muscle due to the absence or reduction in metabolic activity in generating heat after increasing postmortem time, anaerobic metabolism (lactic acid production), and carbonic acid production as carbon dioxide buildup (acidic byproduct of the bacteria).

Within the storage period (Table 3 and Figure 2A), the breast of the whole carcass had a temperature of 3.15 °C compared with the deboned breast meat of 3.94 °C. Temperature was not significantly affected by the change in carcass weight, but it was significantly (*p* < 0.05) affected by the storage period. The temperature of breast muscles stored for 3 days was lower than that of breast muscles stored for 5 days; however, the temperature of both groups did not differ from groups stored for 1 and 7 days. Botka-Paetrak et al. [42] reported that chilling decreased the temperature of the major pectoralis muscle during continuous chilling that lasted for 5 days at 4 °C.

The post chilling pH values presented in Table 3 were not significantly affected by product form or carcass weight but were significantly (*p* < 0.0001) affected by storage period. Similar results were also reported by Abdullah and Matarneh [35] in that pH values were not affected by different carcass weights. Also, Chouliara et al. [43] found that throughout the 9 days of storage of chicken meat, there was a significant reduction in the pH values, which was attributed to the production of lactic acid through lactic acid bacteria (LAB) metabolism. EI Rammouz et al. [44] stored broiler chicken meat for 1, 3, 6, and 9 days at 4 °C and found that as storage time increased, the pH value decreased with the overall pH of 5.85 due to the proteolysis process.

The carcass weight in this experiment did not differ enough to alter breast meat muscle cooking loss. Similar results were obtained by Mehaffey et al. [40]. Abdullah and Matarneh [35] also reported that cooking loss was not affected by carcass weight. There were significant (*p* < 0.0001) differences in cooking loss as affected by storage period (Table 3). Breast meats that were stored for 1 and 7 days had a significantly higher cooking loss than breast meats stored for 3 and 5 days. Differences in cooking loss across various chilling durations are likely attributable to the extent of myofibrillar protein interaction, influencing water retention within the muscle fibers [45]. Table 3 and Figure 2B show the cooking loss percentages as affected by the interaction of product form and storage period (*p* < 0.001). At days 1, 5, and 7, the whole carcass had a higher cooking loss% than those from part-cut deboned breast meat, but at day 3, deboned breast meat had a higher cooking loss% than the whole carcass.

Table 3 showed that WHC% was significantly (*p* < 0.015) affected by product form. The percentage of whole carcasses (33.74) means that the carcasses can hold less water than part-cut deboned breast meat (30.72). This could be related to losses in the form of dripping during storage. In our study, drip loss% was not measured. The WHC% of chilled breast meat decreased significantly (*p* < 0.0461) with increasing storage time (Table 3). Similar WHC% was shown for chilled broiler breast meats that were stored for 1, 3, and 5 days, but 7 days of cold storage had the lowest WHC%. In the present study, temperature and pH decrease during the overall entire cold storage periods, which are the main factors that influenced the reduction in water holding capacity, so the shift in pH and temperature causes a reduction in water holding capacity.

Heavy carcass weight had significantly higher shear force values (3.66 kg/cm^2^) compared with light carcass weight (3.15 kg/cm^2^). The significant difference in tenderness between light and heavy carcass weight may be attributed to the muscle collagen content and connective tissue, which increase with increasing weight [45]. Tenderness increased significantly (*p* < 0.0001) with an increasing storage period (Table 3). This increase may be related to allowing more time for proteolytic enzymes, such as calpains and lysosomal proteases, to work on the cross-bridge between actin and myosin molecules that finally lead to the enzymatic degradation of muscle tissue. The means of shear force values for storage periods 3, 5, and 7 were not statistically different from each other but had significantly lower shear force values than the day 1 storage period. Similar results were reported by Patsias et al. [21], Kriese et al. [46], Yoon, [47], and Gerelt et al. [48].

Eventually, shear force value is affected by an accumulative sequence of events that occur to meat, such as rigor mortis development, proteolysis activities, and complex chemical changes that occur during the carcass cold storage period at a constant refrigerated temperature and holding conditions. Different storage times produce meat with different tenderness; this may be related to differences in the proteolysis rate between storage periods. A significant (*p* < 0.029) product form–storage period interaction was observed for shear force values (Figure 2D). Shear force values were higher for part-cut deboned breast meat than for whole carcasses throughout the entire storage period.

The heavier carcass weight may have a higher pigment concentration (mainly myoglobin), shifting the meat color to darker and redder. Bianchi et al. [7] reported that heavier birds produced darker breast meats (lower lightness value) than lighter birds (higher lightness value), with no difference found in the redness values. Overall, chilled carcasses tended to be lighter, less red, and less yellow after 1 and 3 days of cold storage compared with 5 and 7 days, which were characterized by being darker, being redder, and having more yellowness. Lightness values were significantly decreased with increasing storage time (*p* < 0.0001) (Table 4). Similar results were found in Ab Aziz et al. [6]. Day 3 had the highest lightness value, and day 7 had the lowest value, which refers to less bright meat because meat water molecules at day 3 are tightly bound, causing more light to be reflected and scattered by muscle, or due to more denaturation of sarcoplasmic proteins by a low pH at day 3, which leads to scattering more light [49]. Chouliara et al. [43] found that the L* value decreased progressively up to day 9 of cold storage (4 °C), indicative of the fact that the color of the fresh meat chicken became more dull.

Table 4 also showed that redness values significantly increased with increasing storage period (*p* < 0.0001), whereas day 3 had the lowest redness value and day 7 had the highest value (redder, darker). These results might be due to an increase in storage period, which gives more time for oxygen to combine with available meat pigments, such as myoglobin, and alter the color and appearance of meat, which finally becomes darker.

The lowest yellowness value was observed on day 3, significantly differing from the breast meat stored for 1 and 7 days. Żmijewski et al. [50] stored breast meat for 1, 3, 6, 9, and 12 days at 4 °C and found that the yellowness values were significantly increased during 1 and 3 days and then gradually decreased for 6, 9, and 12 days. Molette et al. [51] studied the effect of extended chilling on b* values during a 9-day storage period at 4 °C and reported that yellowness values increased throughout the entire storage period. In contrast, Chouliara et al. [43] reported that in stored breast meat for 1, 3, 6, and 9 days at 4 °C, the yellowness exhibited similar values during storage. Yellowness values were affected (*p* < 0.05) by the interaction between carcass weight and storage period (Figure 3B). On days 1 and 5, lighter weights had a higher yellowness value than those from heavier weights, whereas on day 3, lighter weights had a lower yellowness value than those from heavier weights, but at day 7, both weights had similar yellowness values. A significant (*p* < 0.01) product form–storage period interaction was also observed for yellowness values (Figure 3A). Whole carcasses stored for 1 and 7 days showed a higher yellowness value than those for part-cut deboned breast meat, whereas part-cut deboned breast meat at days 3 and 5 had a higher yellowness value than whole carcasses. The chroma value was significantly (*p* < 0.001) affected by storage period (Table 4). Chroma values for breast meat stored for 1 day were similar to those stored for 5 days, but they differed from breast muscle stored for 3 and 7 days with the lowest value registered on day 3 and the highest value recorded on day 7. A significant (*p* < 0.01) product form–storage period interaction was shown for chroma values (Figure 3C). The whole carcasses stored for 1 and 7 days showed higher chroma value than those for part-cut deboned breast meat, whereas part-cut deboned breast meat at day 3 and 5 had a higher chroma value than whole carcasses. Chroma values were also significantly affected (*p* < 0.05) by the interaction between carcass weight and storage period (Figure 3D). On days 1 and 5, lighter weights had higher yellowness values than those from heavier weights, whereas at day 3, lighter weights had a lower yellowness value than those from heavier weight, but on day 7, both weights had similar yellowness values. Hue value was significantly decreased with storage period (*p* < 0.0001), whereas breast meat stored for 1 and 3 days and for 5 and 7 days had similar values. Pectoralis major muscle chroma and hue angles were not significantly affected by carcass weight during post-chilling times [35]. Crude protein (*p* < 0.05) and ash (*p* < 0.05) were significantly higher in light weight carcasses compared with heavy weight carcasses. The mean values obtained for light and heavy weights were 21.30 and 21.06, respectively, for crude protein. Ether extract was significantly higher (*p* < 0.0001) for heavy weights (1.0%) than light weights (0.70%). Heavier carcasses typically have higher fat content and lower percentages of crude protein and water [52]. The results for crude protein showed that the breast meat stored for 1, 3, and 5 days did not differ significantly. However, breast meat stored for 7 days of cold storage showed significantly higher crude protein compared with 1, 3, and 5 days. Mean values of breast meat chemical composition for day 1 were 21.03 and 0.77; for day 3 they were 21.03 and 0.75; for day 5 they were 21.12 and 0.85; and for day 7 they were 21.55 and 1.03 for crude protein and ether extract, respectively. These findings may be due to proteolysis mechanisms (protease system).

Raw poultry meat sold in supermarkets may be contaminated with various microorganisms. This is of great interest to consumers and producers due to the high risk of bacterial contamination and the spread of foodborne diseases [16,17,53,54,55]. Capita et al. [56] found that the APC of vacuum-packaged ostrich meat was 4.9, 6.4, 7.1, and 8.0 log_10_ CFU/g, respectively, after 0, 3, 6, and 9 days of cold storage at 4 °C.

A significant reduction in the viability of aerobic bacteria was noted after the first day of chilled storage. Unpredictably, the APC was lower in BH and BL (3.38 and 3.63 log_10_ CFU/g, respectively) than in WH and WL (4.19 and 4.03 log_10_ CFU/g, respectively). The APC increased in whole carcasses more than in deboned breast meat (Figure 4A). The reduction in APC could be explained by the idea that part of the contaminated bacteria was mesophilic in nature, which means it loses its ability to multiply at lower temperatures. In addition, the lower APC of the deboned breast compared to the whole carcass may be explained by the lower surface area, since the internal parts of deboned cuts will contain a lower number of bacteria compared to the surface. After day one, APC continued to increase until reaching the maximal recommended limit of 6 log_10_ CFU/g. The APC was 5.85 and 6.32 log_10_ CFU/g in BH and BL, respectively, and 6.20 and 6.16 log_10_ CFU/g in WH and WL, respectively. At this level of APC, the signs of spoilage started to appear since a slight off-odor and sliminess were noted.

As cold storage time increased, the psychrotrophic count increased significantly from 3.13 and 3.41 log_10_ CFU/g in BH and BL to reach 7.47 and 7.57 log_10_ CFU/g, respectively. Similarly, the initial psychrotrophic count increased from 4.02 and 3.73 log_10_ CFU/g in WH and WL to 7.45 and 7.42 log_10_ CFU/g, respectively, after 7 days.

Capita et al. [56] found that psychrotrophic counts of vacuum-packaged ostrich meat were 3.6, 5.4, 6.4, and 7.4 log_10_ CFU/g, respectively, after 0, 3, 6, and 9 days of cold storage. Figure 4C presents the proteolytic count (PLC) as affected by different cold storage times (1, 3, 5, and 7 days). The initial proteolytic count of BH and BL increased from 4.75 and 4.81 log_10_ CFU/g to 7.07 and 7.29 log_10_ CFU/g, respectively, after 7 days of cold storage. Similarly, the initial proteolytic count of WH and WL increased from 4.86 and 4.82 to 7.20 and 6.58 log_10_ CFU/g, respectively, after 7 days of cold storage. Since the concentrations of fermentable carbohydrates are very low in broiler carcasses, spoilage bacteria will start to metabolize other nutrients as a source of energy. Lipolytic and proteolytic bacteria are considered among the most important psychrotrophic spoilage bacteria in meat carcasses since their growth could change sensory characteristics such as color and flavor [57].

The coliform count for both whole carcasses and deboned breast meat was close to the seven maximal recommended limits as specified by Jordanian standards (6 log_10_ CFU/g). Carroll and Alvarado [58] found that coliform counts of marinated breast meat increased with storage period to reach 4.8 log_10_ CFU/g after 9 days.

In the present study, the whole carcasses and deboned breast meat of broiler chicken carcasses have a refrigerated shelf life between 3 and 5 days of cold storage based on the microbiological quality, which should not exceed 6 log_10_ CFU/g.

## 5. Conclusions

It appears that product form, carcass weight, and storage period play a major role in the overall quality of poultry meat (bacteriological and nutritive quality). Based on our research results, as the storage period increased, L*, tenderness, cooking loss, water holding capacity, post-chilling temperature, and pH decreased, but a* increased. As carcass weight increased, L* and water holding capacity decreased, but a* and tenderness increased. Whole carcass meat was characterized by higher L*, pH, cooking loss, and water holding capacity compared with part-cut deboned breast meat. Therefore, storing meat as a whole has a greater positive impact on quality as opposed to part-cut deboned breast meat. There are significant differences in protein and ether extracts by each carcass weight category and each storage period. As storage time increased, aerobic plate count (APC), psychrotrophic count (PTC), proteolytic count (PLC), lipolytic count (LLC), and colonic count (CC) increased significantly. However, based on the meat quality and microbiology evaluation, the maximum freshness limit and shelf life for refrigerated chicken meat are 3 to 5 days. In conclusion, the results of the present study are considered to be evidence of the effect of cold storage on microbial quality and physicochemical properties. This emphasizes the need to adopt good manufacturing practices in handling raw chicken and the use of effective cooking temperatures to ensure the destruction of pathogenic microorganisms.

## Figures and Tables

**Table 1:** Starter and finisher diet composition.

Ingredients (%)	Starter	Finisher
Yellow corn	58	62.5
Soybean meal	28	23
Concentrate	10	10
Vegetable oil	3	3.5
Di-Ca-Phosphate	0.3	0.3
Limestone	0.5	0.5
Methionine	0.1	0.1
Lysine	0.1	0.1
Total	100	100
**Nutrient Composition**		
ME, kca/kg *	3180	3220
Crude protein	23.00	21.00
Lysine *	1.30	1.20
Methionine *	0.55	0.50
Calcium *	0.80	0.75
Available phosphorus *	0.50	0.50

* calculated values.

**Table 2:** Least-squares means for broiler carcass postmortem temperature and pH.

Item	Temperature (°C)	pH
Carcass Weight (g)		
Light Weight (1100 ± 50 g)	6.19	6.06
Heavy Weight (1400 ± 50 g)	6.63	6.09
S.E	0.19	0.04
*p*-value	NS	NS
Postmortem Time (hour)		
1 h	11.22 ^a^	6.18 ^a^
2 h	7.89 ^b^	6.14 ^a^
3 h	3.34 ^c^	6.01 ^ab^
4 h	3.18 ^c^	5.96 ^b^
S.E	0.27	0.06
*p*-value	0.0001	0.037
Interactions		
CW × PT	NS	NS

^a,b,c^ means within the same column with different superscripts differ according to the indicated level of significance within each main effect. NS = non-significant.

**Table 3:** Least-squares means for broiler breast meat post-chilling temperature and pH and for cooking loss (CL), water holding capacity (WHC), and Warner–Bratzler shear force (WBSF) as affected by product form (PF), carcass weight (CW), and storage period (SP).

Item	N	Temperature (°C)	pH	CL (%)	WHC (%)	WBSF (Kg/cm^2^)
Product Form						
Whole Carcass	64	3.15 ^b^	5.63	38.01 ^a^	33.74 ^a^	3.14 ^b^
Deboned Breast Meat	64	3.94 ^a^	5.59	35.43 ^b^	30.72 ^b^	3.67 ^a^
S.E		0.12	0.01	0.52	0.95	0.10
*p*-value		0.0001	NS	0.0005	0.0122	0.0004
Carcass Weight (g)						
Light Weight (1100 ± 50 g)	64	3.57	5.61	37.10	34.03 ^a^	3.15 ^b^
Heavy Weight (1400 ± 50 g)	64	3.52	5.61	36.35	30.43 ^b^	3.66 ^a^
S.E		0.12	0.01	0.51	0.84	0.10
*p*-value		NS	NS	NS	0.003	0.0005
Storage Period (Day)						
1 d	32	3.66 ^ab^	5.81 ^a^	39.56 ^a^	33.50 ^a^	5.55 ^a^
3 d	32	3.25 ^a^	5.66 ^b^	34.11 ^b^	32.84 ^a^	2.80 ^b^
5 d	32	3.85 ^b^	5.57 ^c^	33.60 ^b^	33.27 ^a^	2.62 ^b^
7 d	32	3.42 ^ab^	5.41 ^d^	39.63 ^a^	29.32 ^b^	2.66 ^b^
S.E		0.17	0.02	0.73	1.18	0.14
*p*-value		0.033	0.0001	0.0001	0.0461	0.0001
Interactions						
PF × CW		NS	NS	NS	0.0008	NS
PF × SP		0.0001	NS	0.001	NS	0.029
CW × SP		NS	NS	NS	NS	NS
PF × CW × SP		NS	NS	NS	NS	NS

^a,b,c,d^ means within the same column with different superscripts differ according to the indicated level of significance within each main effect. NS = non-significant. N = number of representative samples for each treatment. CL% = cooking loss percentage. WHC% = water holding capacity percentage. WBSF = Warner–Bratzler shear force in kilogram per centimeters squared. The temperature is Celsius degrees.

**Table 4:** Least-squares means for broiler breast meat color values as affected by product form (PF), carcass weight (CW), and storage period (SP).

Item	N	L*	a*	b*	Chroma	Hue
Product Form						
Whole Carcass	64	52.84 ^a^	5.22	15.96	16.82	71.87
Deboned Breast Meat	64	51.71 ^b^	5.18	16.10	16.94	71.91
S.E		0.35	0.06	0.21	0.20	0.30
*p*-value		0.022	NS	NS	NS	NS
Carcass Weight (g)						
Light Weight (1100 ± 50 g)	64	52.98 ^a^	5.16	16.13	16.96	72.27
Heavy Weight (1400 ± 50 g)	64	51.57 ^b^	5.24	15.94	16.80	71.50
S.E		0.35	0.06	0.21	0.20	0.30
*p*-value		0.005	NS	NS	NS	NS
Storage Period (Day)						
1 d	32	54.19 ^b^	4.70 ^d^	16.39 ^a^	17.06 ^ac^	73.85 ^a^
3 d	32	59.67 ^a^	4.37 ^c^	15.40 ^b^	16.03 ^b^	73.63 ^a^
5 d	32	49.40 ^c^	5.70 ^b^	15.77 ^ab^	16.78 ^ab^	69.93 ^b^
7 d	32	45.84 ^d^	6.03 ^a^	16.57 ^a^	17.65 ^c^	70.15 ^b^
S.E		0.49	0.08	0.29	0.28	0.42
*p*-value		0.0001	0.0001	0.021	0.001	0.0001
Interactions						
PF × CW		NS	NS	NS	NS	NS
PF × SP		NS	NS	0.004	0.003	NS
CW × SP		NS	NS	0.029	0.041	NS
PF × CW × SP		NS	NS	NS	NS	NS

^a,b,c,d^ means within the same column with different superscripts differ according to the indicated level of significance within each main effect. NS = non-significant. N = number of representative samples for each treatment. L* = lightness/brightness value. a* = redness value. b* = yellowness value.

**Table 5:** Least-squares means for broiler breast meat chemical composition% (on fresh basis) as affected by storage period (SP) and carcass weight (CW).

Item	Moisture%	Crude Protein%	Ether Extract%	Ash%
Carcass Weight (g)				
Light weight (1100 ± 50 g)	74.79	21.30 ^a^	0.70 ^b^	1.13 ^a^
Heavy weight (1400 ± 50 g)	75.15	21.06 ^b^	1.00 ^a^	1.07 ^b^
S.E	0.15	0.083	0.04	0.02
*p*-value	NS	0.046	0.0001	0.049
Storage Period (Day)				
1 d	75.08	21.03 ^a^	0.77 ^a^	1.09
3 d	75.06	21.03 ^a^	0.75 ^a^	1.07
5 d	75.15	21.12 ^a^	0.85 ^b^	1.14
7 d	74.59	21.55 ^b^	1.03 ^c^	1.10
S.E	0.21	0.12	0.05	0.03
*p*-value	NS	0.011	0.004	NS
Interactions				
CW × SP	NS	0.040	NS	NS

^a,b,c^ means within the same column with different superscripts differ according to the indicated level of significance within each main effect. NS = non-significant.

## Data Availability

The original contributions presented in this study are included in the article. Further inquiries can be directed to the corresponding author.

## References

[1] Osaili T.M., Hasan F., Dhanasekaran D.K., Arasudeen A., Ismail L.C., Hasan H., Hashim M., Faris M.A.E., Radwan H., Naja F. (2024). Preservative effect of pomegranate-based marination with β-resorcylic acid and cinnamaldehyde on the microbial quality of chicken liver. Poult. Sci..

[2] Whitton C., Bogueva D., Marinova D., Phillips C.J.C. (2021). Are we approaching peak meat consumption? Analysis of meat consumption from 2000 to 2019 in 35 countries and its relationship to gross domestic product. Animals.

[3] Petracci M., Bianchi M., Mudalal S., Cavani C. (2013). Functional ingredients for poultry meat products. Trends Food Sci. Technol..

[4] Dal Bosco A., Mattioli S., Cartoni Mancinelli A., Cotozzolo E., Castellini C. (2021). Extensive rearing systems in poultry production: The right chicken for the right farming system. A review of twenty years of scientific research in Perugia University, Italy. Animals.

[5] Mancinelli A.C., Mattioli S., Twining C., Dal Bosco A., Donoghue A.M., Arsi K., Angelucci E., Chiattelli D., Castellini C. (2022). Poultry meat and eggs as an alternative source of n-3 long-chain polyunsaturated fatty acids for human nutrition. Nutrients.

[6] Ab Aziz M.F., Hayat M.N., Kaka U., Kamarulzaman N.H., Sazili A.Q. (2020). Physico-chemical characteristics and microbiological quality of broiler chicken pectoralis major muscle subjected to different storage temperature and duration. Foods.

[7] Bianchi M., Petracci M., Cavani C. (2006). The influence of genotype, market live weight, transportation, and holding conditions prior to slaughter on broiler breast meat color. Poult. Sci..

[8] Mir N.A., Rafiq A., Kumar F., Singh V., Shukla V. (2017). Determinants of broiler chicken meat quality and factors affecting them: A review. J. Food Sci. Technol..

[9] Park S.Y., Byeon D.S., Kim G.W., Kim H.Y. (2021). Carcass and retail meat cuts quality properties of broiler chicken meat based on the slaughter age. J. Anim. Sci. Technol..

[10] Gharaibeh M.H., Khalifeh M.S., Nawasreh A.N., Hananeh W.M., Awawdeh M.S. (2021). Assessment of immune response and efficacy of essential oils application on controlling necrotic enteritis induced by Clostridium perfringens in broiler chickens. Molecules.

[11] Fung F., Wang H.S., Menon S. (2018). Food safety in the 21st century. Biomed. J..

[12] Castro V.S., Figueiredo E., McAllister T., Stanford K. (2022). Farm to fork impacts of super-shedders and high-event periods on food safety. Trends Food Sci. Technol..

[13] Ovuru K.F., Izah S.C., Ogidi O.I., Imarhiagbe O., Ogwu M.C. (2024). Slaughterhouse facilities in developing nations: Sanitation and hygiene practices, microbial contaminants and sustainable management system. Food Sci. Biotechnol..

[14] Gourama H. (2020). Foodborne pathogens. Food Safety Engineering.

[15] Kamboj S., Gupta N., Bandral J.D., Gandotra G., Anjum N. (2020). Food safety and hygiene: A review. Int. J. Chem. Stud..

[16] Jaradat Z., Khataybeh B., Al Ghzawi A.M., Ababneh Q., Al Nabusli A. (2022). Molecular identification of major bacteria in honey and the effect of microwave treatment on its microbial quality and antibacterial activity. AIMS Agric. Food.

[17] Jaradat Z., Aldakil H., Tadros M., Alboom M., Khataybeh B. (2023). Rhizoctonia solani AG-3PT is the major pathogen associated with potato stem canker and black scurf in Jordan. AIMS Agric. Food.

[18] Katiyo W., de Kock H.L., Coorey R., Buys E.M. (2020). Sensory implications of chicken meat spoilage in relation to microbial and physicochemical characteristics during refrigerated storage. LWT.

[19] Rouger A., Tresse O., Zagorec M. (2017). Bacterial contaminants of poultry meat: Sources, species, and dynamics. Microorganisms.

[20] Boubendir S., Arsenault J., Quessy S., Thibodeau A., Fravalo P., Thériault W.P., Fournaise S., Gaucher M.L. (2021). Salmonella contam-ination of broiler chicken carcasses at critical steps of the slaughter process and in the environment of two slaughter plants: Prevalence, genetic profiles, and association with the final carcass status. J. Food Prot..

[21] Patsias A., Badeka A.V., Savvaidis I.N., Kontominas M.G. (2008). Combined effect of freeze chilling and MAP on quality parameters of raw chicken fillets. Food Microbiol..

[22] Wang H., Qin X., Li X., Wang X., Gao H., Zhang C. (2020). Changes in the microbial communities of air-and water-chilled yellow-feathered broilers during storage at 2 °C. Food Microbiol..

[23] Adley C.C., Ryan M.P. (2016). The nature and extent of foodborne disease. Antimicrobial Food Packaging.

[24] Sing A. (2023). Zoonoses: Infections Affecting Humans and Animals.

[25] The World Bank The World by Income and Region. https://datatopics.worldbank.org/world-development-indicators/the-world-by-income-and-region.html.

[26] Alaboudi A.R., Malkawi I.M., Osaili T.M., Abu-Basha E.A., Guitian J. (2020). Prevalence, antibiotic resistance and genotypes of Cam-pylobacter jejuni and *Campylobacter coli* isolated from chickens in Irbid governorate, Jordan. Int. J. Food Microbiol..

[27] Hantash T., Apenteng O.O., Alhawajreh A., Nofal M., Atyyah M., Zaghal R., Vigre H. (2021). Zoonotic Pathogens in Frozen Red and Poultry Meat imports to Jordan. in the period 2015–2019: Salmonella Typhimurium, Salmonella Enteritidis, Listeria monocytogenes and *E. coli* O157: H7. Curr. Res. Microbiol. Infect..

[28] Roussan D.A., Haddad R., Khawaldeh G. (2008). Molecular survey of avian respiratory pathogens in commercial broiler chicken flocks with respiratory diseases in Jordan. Poult. Sci..

[29] Ibrahim R.A., Cryer T.L., Lafi S.Q., Basha E.A., Good L., Tarazi Y.H. (2019). Identification of *Escherichia coli* from broiler chickens in Jordan, their antimicrobial resistance, gene characterization and the associated risk factors. BMC Vet. Res..

[30] Nimri L., Al-Dahab F.A., Batchoun R. (2014). Foodborne bacterial pathogens recovered from contaminated shawarma meat in northern Jordan. J. Infect. Dev. Ctries..

[31] Gharaibeh M.H., Al Sheyab S.Y., Lafi S.Q., Etoom E.M. (2024). Risk factors associated with mcr-1 colistin-resistance gene in *Escherichia coli* broiler samples in northern Jordan. J. Glob. Antimicrob. Resist..

[32] Osaili T.M., Alaboudi A.R., Al-Akhras R.R. (2012). Prevalence and antimicrobial susceptibility of *Campylobacter* spp. in live and dressed chicken in Jordan. Foodborne Pathog. Dis..

[33] Gharaibeh S., Al Rifai R., Al-Majali A. (2010). Molecular typing and antimicrobial susceptibility of Clostridium perfringens from broiler chickens. Anaerobe.

[34] Osaili T.M., Alaboudi A.R., Nesiar E.A. (2011). Prevalence of *Listeria* spp. and antibiotic susceptibility of Listeria monocytogenes isolated from raw chicken and ready-to-eat chicken products in Jordan. Food Control.

[35] Abdullah A.Y., Matarneh S.K. (2010). Broiler performance and the effects of carcass weight, broiler sex, and postchill carcass aging duration on breast fillet quality characteristics. J. Appl. Poult. Res..

[36] National Research Council (1994). Nutrient Requirements of Poultry.

[37] Abdullah A.Y., Obeidat B.S., Khataybeh B.T. (2024). Effects of Feeding Prosopis Juliflora Pods on Growth Performance, Nutrient In-take, Digestibility, and Carcass Characteristics of Black Goat Kids Fed Finishing Diets. Jordan J. Agric. Sci..

[38] Official Methods of Analysis (1990). Association of Official Analytical Chemist.

[39] SAS Institute Inc (2000). SAS/STAT User’s Guide, 6..

[40] Mehaffey J.M., Pradhan S.P., Meullenet J.F., Emmert J.L., McKee S.R., Owens C.M. (2006). Meat quality evaluation of minimally aged broiler breast fillets from five commercial genetic strains. Poult. Sci..

[41] Abdullah A.Y., Muwalla M.M., Maharmeh H.O., Matarneh S.K., Ishmais M.A. (2010). Effects of strain on performance, and age at slaughter and duration of post-chilling aging on meat quality traits of broiler. Asian-Australas. J. Anim. Sci..

[42] Botka-Petrak K., Vidaček S., Petrak T., Medić H. (2005). Influence of combined, continuous chilling on physical and chemical prop-erties of white and red chicken muscles. Vet. Arh..

[43] Chouliara E., Karatapanis A., Savvaidis I.N., Kontominas M.G. (2007). Combined effect of oregano essential oil and modified atmosphere packaging on shelf-life extension of fresh chicken breast meat, stored at 4 °C. Food Microbiol..

[44] El Rammouz R., Babilé R., Fernandez X. (2004). Effect of ultimate pH on the physicochemical and biochemical characteristics of turkey breast muscle showing normal rate of postmortem pH fall. Poult. Sci..

[45] Purslow P.P., Oiseth S., Hughes J., Warner R.D. (2016). The structural basis of cooking loss in beef: Variations with temperature and ageing. Food Res. Int..

[46] Kriese P.R., Soares A.L., Guarnieri P.D., Prudencio S.H., Ida E.I., Shimokomaki M. (2007). Biochemical and sensorial evaluation of intact and boned broiler breast meat tenderness during ageing. Food Chem..

[47] Yoon K.S. (2003). Effect of gamma irradiation on the texture and microstructure of chicken breast meat. Meat Sci..

[48] Gerelt B., Ikeuchi Y., Nishiumi T., Suzuki A. (2002). Meat tenderization by calcium chloride after osmotic dehydration. Meat Sci..

[49] Fletcher D.L. (2002). Poultry meat quality. World’s Poult. Sci. J..

[50] Żmijewski T., Kwiatkowska A., Cierach M. (2009). The effect of cold storage on the color of venison. Pol. J. Nat. Sci..

[51] Molette C., Remignon H., Babile R. (2005). Modification of glycolyzing enzymes lowers meat quality of turkey. Poult. Sci..

[52] Bianchi M., Petracci M., Sirri F., Folegatti E., Franchini A., Meluzzi A. (2007). The influence of the season and market class of broiler chickens on breast meat quality traits. Poult. Sci..

[53] Afify E.S., Shaltout F.A., Zakaria I.M. (2020). Aerobic plate count of contaminants and molecular characterization of *Eschereichia coli* in raw chicken meat in Ismailia, Egypt. J. Vet. Healthc..

[54] Dourou D., Doulgeraki A.I., Vitsou-Anastasiou S., Argyri A.A., Chorianopoulos N.G., Nychas G.J., Tassou C.C. (2023). Deciphering the growth responses and genotypic diversity of bioluminescent Photobacterium phosphoreum on chicken meat during aerobic refrigerated storage. Int. J. Food Microbiol..

[55] Siluma B.J., Kgatla E.T., Nethathe B., Ramashia S.E. (2023). Evaluation of meat safety practices and hygiene among different butcheries and supermarkets in Vhembe District, Limpopo Province, South Africa. Int. J. Environ. Res. Public Health.

[56] Capita R., Díaz-Rodríguez N., Prieto M., Alonso-Calleja C. (2006). Effects of temperature, oxygen exclusion, and storage on the microbial loads and pH of packed ostrich steaks. Meat Sci..

[57] Wang GuangYu W.G., Wang HuHu W.H., Han YiWei H.Y., Xing Tong X.T., Ye KePing Y.K., Xu XingLian X.X., Zhou GuangHong Z.G. (2017). Evaluation of the spoilage potential of bacteria isolated from chilled chicken *in vitro* and *in situ*. Food Microbiol..

[58] Carroll C.D., Alvarado C.Z. (2008). Comparison of air and immersion chilling on meat quality and shelf life of marinated broiler breast fillets. Poult. Sci..

